# Goiter, thyroid facial involvement and eye disease in the paintings of Jan Gossaert (c. 1478–1532)

**DOI:** 10.1007/s40618-023-02104-5

**Published:** 2023-05-06

**Authors:** H. Ashrafian

**Affiliations:** Institute of Global Health Innovation, The Department of Surgery and Cancer, Imperial College London, 10th Floor Queen Elizabeth the Queen Mother (QEQM) Building, St. Mary’s Hospital, Praed Street, London, W2 1NY UK

The genius of the Renaissance was sustained through the dissemination of artistic innovations from its multiple centres. Amongst these, Jan Gossaert (c. 1478–1532) also called Jan Mabuse, was a notable champion who likely originated in the region of the French-Belgian border (medieval Hainault) or the province of Utrecht in the Netherlands and travelled to Rome in 1508/9 before settling in the Netherlands to lead the artistic style of *Romanism*. This era corresponds to that of the High Renaissance in Italy so that his work is considered as representing the virtuosity of that era in Northwestern Europe.

**Fig. 1:**
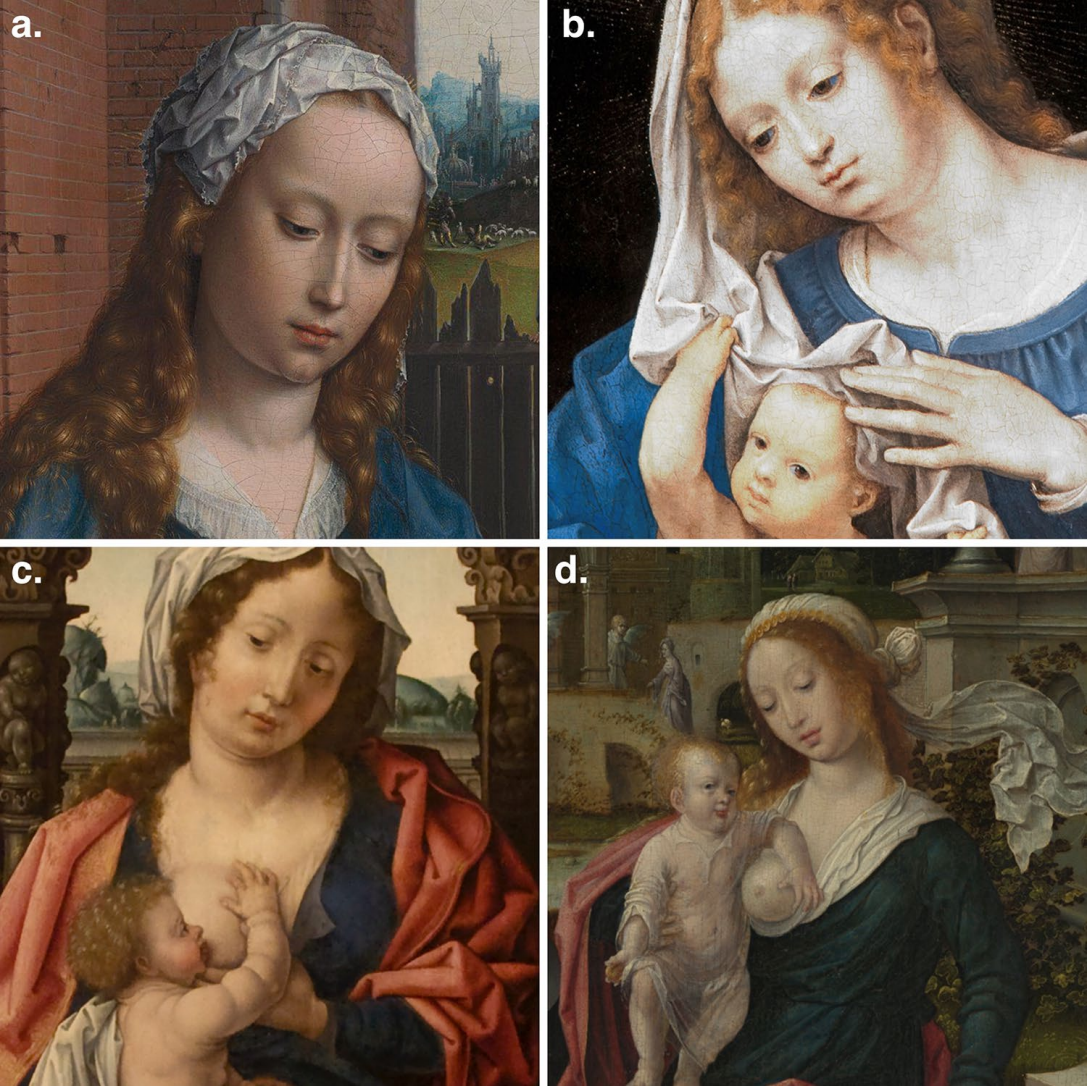
Jan Gossaert (also called Jan Mabuse), reproduced icons which are details of the original paintings. **a** Adoration of the Magi (1510–15) © National Gallery, London, UK. **b** Madonna and Child Playing with the Veil (c. 1520) © Mauritshuis, The Hague, Netherlands. **c** Virgin and Child (1508–10) © Calouste Gulbenkian Museum, Lisbon, Portugal. **d** The Holy Family (1507/8) © Getty Center, Los Angeles, USA

The Italian Renaissance catalogues multiple examples of goiter likely deriving from iodine deficiency and autoimmunity captured by the artistic realism of the era [1, 2]. I now note examples of Goiters in several paintings by Jan Gossaert (Fig. 1a–d). Just as in the Italian Renaissance these can originate from iodine deficiency and autoimmunity in Northwestern Europe. Similarly, the presence of goiter is presented through classic neck fullness and da Vinci Sign, with loss or shallowing of the suprasternal notch recess or the Botticelli Sign where cranio-cervical neck flexion accentuates thyroid enlargement [1, 2].

In these paintings the goiter is associated with Sign of Hertoghe (Queen Anne’s sign) [3] by loss of the lateral eyebrow (Fig. 1a–c), facial erythema (Fig. 1a, c), and degrees of thyroid eye disease and ptosis (Fig. 1a–d.). Whilst these images may be stylized, they nonetheless may represent underlying pathology in the individuals represented. One of the paintings has figures with blue-tinged sclera, notable in the Madonna (Fig. 1b.). This may be purely artistic style, though could also represent hyperpigmentation conditions (including melanosis, melanoma, melanocytosis, nevi or Addison’s Disease) or known Alkaptonuria and hypothyroidism [4] presenting with blue sclera through ocular ochronosis.

This work emphasizes the consistency of artistic realism across multiple geographies to capture thyroid disease in its multiple representations in Southern and Northwestern Europe and presents the international spread of genius masters and their innovative artistic techniques at the key inflection point of modern civilization.
